# Supplementary material for: Human eukaryotic initiation factor 4G directly binds the 40S ribosomal subunit to promote efficient translation
Source: J Biol Chem. 2024 Apr 1;300(5):107242. doi: 10.1016/j.jbc.2024.107242 (PMC11063902; doi:10.1016/j.jbc.2024.107242)
Supplement: Supporting Figures S1–S3 and Tables S1 and S2 [file mmc1.docx]

Supporting Information:

Human eukaryotic initiation factor 4G directly binds the 40S ribosomal subunit to promote efficient translation

Nancy Villa and Christopher Fraser

**Supplementary Figure 1**

**
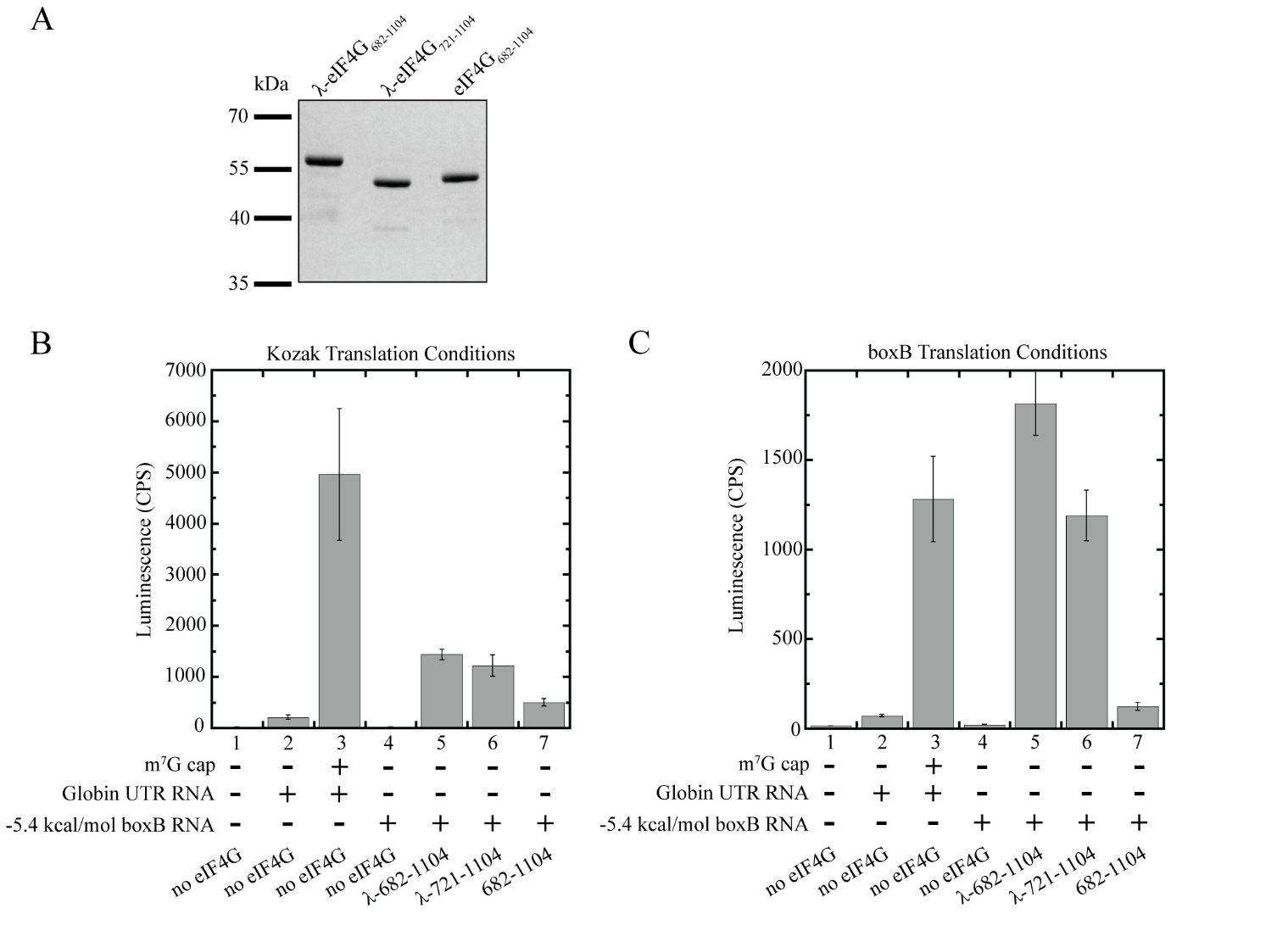
**

**Figure S1. boxB translation assay is active and selective for λ-eIF4G mediated translation. (A)** Coomassie stained gel of λ-tagged and untagged eIF4G constructs used to supplement translation reactions in RRL. **(B)** Translation in RRL using “Kozak” buffer conditions (see *Experimental Procedures*), which are optimized for m^7^G capped reporter translation. **(C)** Translation in RRL using “boxB” buffer conditions (see *Experimental Procedures*), which efficiently translates boxB reporter RNAs only upon addition of λ-eIF4G constructs capable of recruiting a ribosome. Translation reactions show the average of 3 experiments ± SEM. Bar graphs show raw translation data in Luminescence Counts per Second (CPS).

Supporting Information:

Human eukaryotic initiation factor 4G directly binds the 40S ribosomal subunit to promote efficient translation

Nancy Villa and Christopher Fraser

**Supplementary Figure 2**


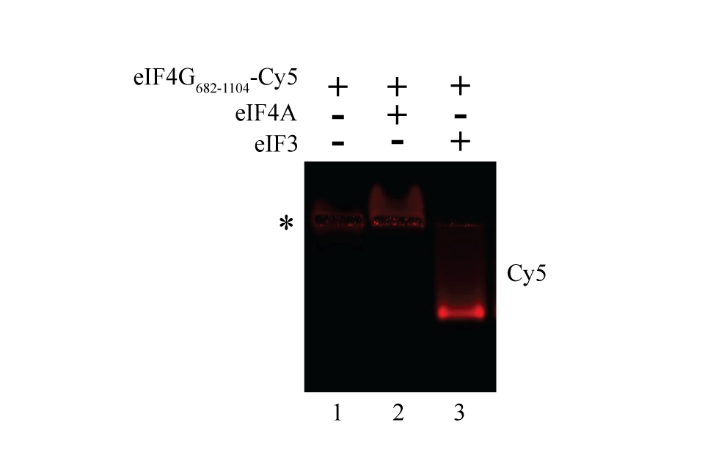


**Figure S2. Native gel electrophoresis controls for eIF4G_682-1104_-Cy5.** eIF4G_682-1104_-Cy5 largely remains in the well during native gel electrophoresis when free or bound to eIF4A (lanes 1 and 2). Upon addition of eIF3, eIF4G_682-1104_-Cy5 enters the gel and comigrates to form a distinct band indicating complex formation (lane 3). Location of the wells is marked by an asterisk (*).

Supporting Information:

Human eukaryotic initiation factor 4G directly binds the 40S ribosomal subunit to promote efficient translation

Nancy Villa and Christopher Fraser

**Supplementary Figure 3**


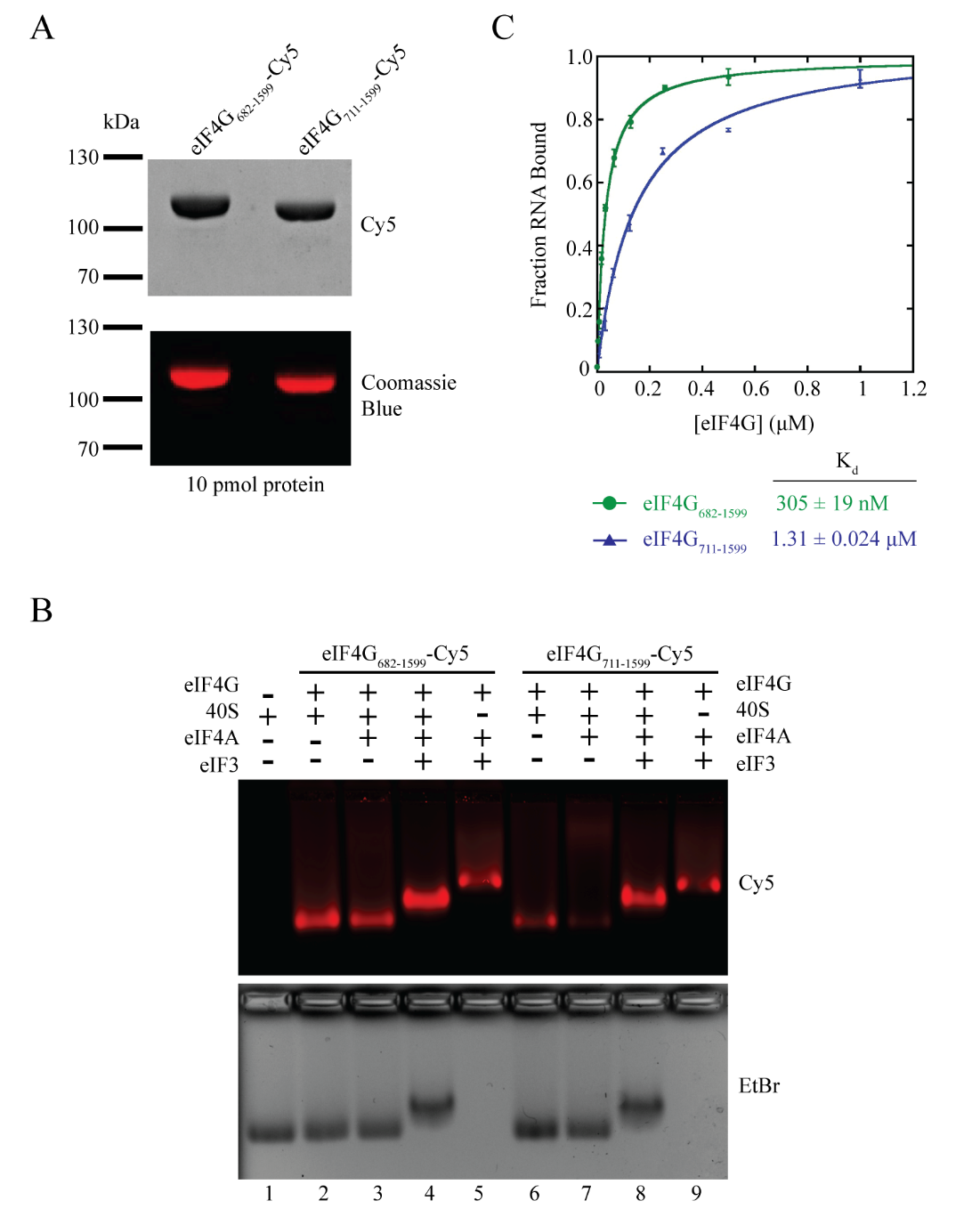


**Figure S3. eIF4G_682-1599_ binds the 40S subunit. (A)** Cysteine-free eIF4G_682-1599_ and eIF4G_711-1599_ constructs each have a single cysteine mutation at residue E711 for site specific modification with Cy5. Following modification, 10 pmol of each protein was separated by SDS PAGE, imaged to visualize the Cy5 fluorophore then stained with coomassie blue to confirm purity of each sample and equal levels of Cy5 modification efficiency. (**B**) 40S subunits (300 nM), eIF4A (2 μM), eIF3 (600 nM), 0.5 mM ATP, and eIF4G-Cy5 (100 nM) were incubated together for 10 min on ice followed by 5 min at 30 °C in various combinations, then separated by native gel electrophoresis to visualize complex formation. Gels were imaged to visualize the Cy5 fluorophore on eIF4G then stained with ethidium bromide to directly observe the 40S ribosomal subunit. Compare eIF4G_682-1599_ complex formation (lanes 2-5) to the RNA binding truncation eIF4G_711-1599_ (lanes 6-9). (**C**) Fluorescence polarization assays are used to determine the equilibrium dissociation constant (K_d_) of the eIF4G•RNA interaction using a 42 nucleotide 3′-end fluorescein-labeled RNA. Fraction of RNA bound by eIF4G_682-1599_ (green), or eIF4G_711-1599_ (blue). Data are the average of at least 3 trials and error bars represent the SEM.

Supporting Information:

Human eukaryotic initiation factor 4G directly binds the 40S ribosomal subunit to promote efficient translation

Nancy Villa and Christopher Fraser

**Supplementary Table 1**


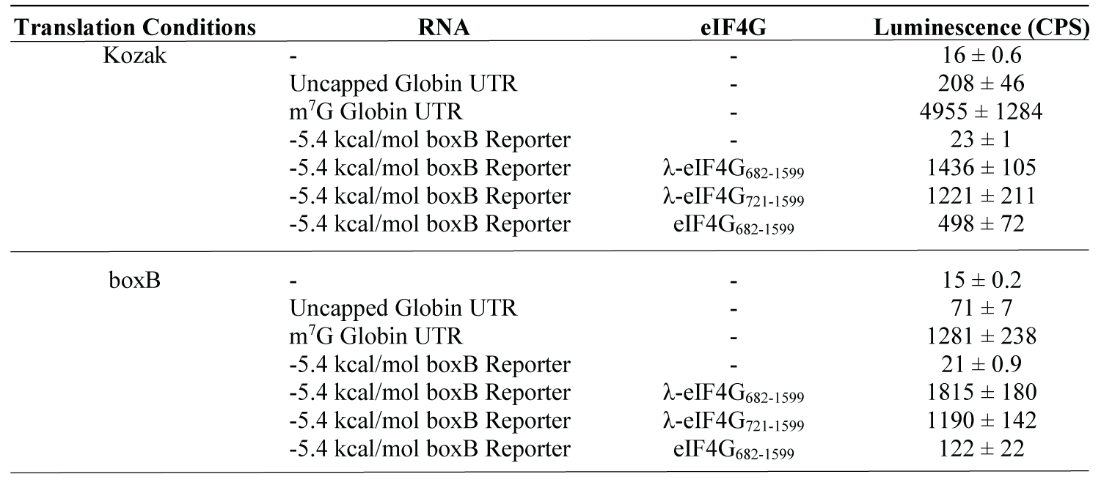


**Table S1. Summary of translation data for Fig. S1.**

Supporting Information:

Human eukaryotic initiation factor 4G directly binds the 40S ribosomal subunit to promote efficient translation

Nancy Villa and Christopher Fraser

**Supplementary Table 2**

**
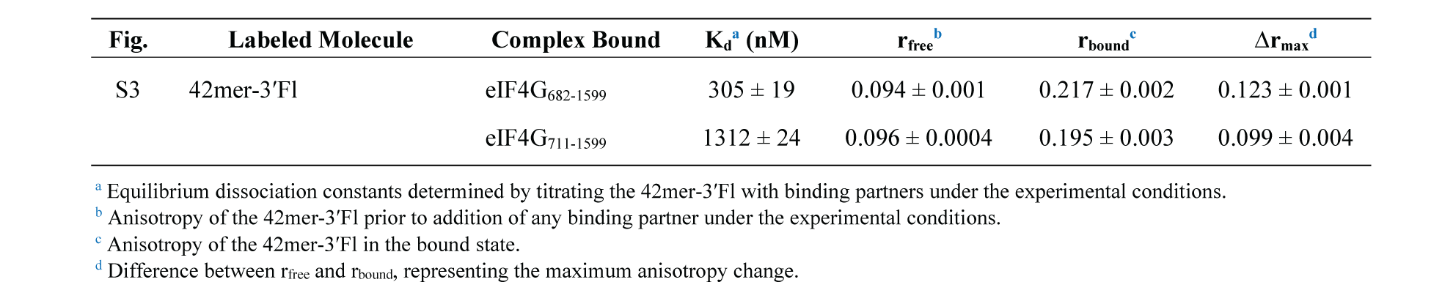
**

**Table S2. Summary of equilibrium binding parameters for Fig. S3.**
